# An Investigation of the Diversity of Strains of Enteroaggregative *Escherichia coli* Isolated from Cases Associated with a Large Multi-Pathogen Foodborne Outbreak in the UK

**DOI:** 10.1371/journal.pone.0098103

**Published:** 2014-05-20

**Authors:** Timothy J. Dallman, Marie A. Chattaway, Lauren A. Cowley, Michel Doumith, Rediat Tewolde, David J. Wooldridge, Anthony Underwood, Derren Ready, John Wain, Kirsty Foster, Kathie A. Grant, Claire Jenkins

**Affiliations:** 1 Gastrointestinial Bacteria Reference Unit, Public Health England, London, United Kingdom; 2 Antimicrobial Resistance and Healthcare Associated Infections Reference Unit, Public Health England, London, United Kingdom; 3 Applied Laboratory and Bio- Informatics Unit, Public Health England, London, United Kingdom; 4 Department for Bio-analysis and Horizon Technologies, Next Generation Sequencing, Public Health England, London, United Kingdom; 5 Public Health Laboratory London, Barts & The London Hospital Trust, London, United Kingdom; 6 Innovation Centre, University of East Anglia, Norwich, United Kingdom; 7 North East Public Health England Centre, Newcastle upon Tyne, United Kingdom; Institut National de la Recherche Agronomique, France

## Abstract

Following a large outbreak of foodborne gastrointestinal (GI) disease, a multiplex PCR approach was used retrospectively to investigate faecal specimens from 88 of the 413 reported cases. Gene targets from a range of bacterial GI pathogens were detected, including *Salmonella* species, *Shigella* species and Shiga toxin-producing *Escherichia coli*, with the majority (75%) of faecal specimens being PCR positive for *aggR* associated with the Enteroaggregative *E. coli* (EAEC) group. The 20 isolates of EAEC recovered from the outbreak specimens exhibited a range of serotypes, the most frequent being O104:H4 and O131:H27. None of the EAEC isolates had the Shiga toxin (stx) genes. Multilocus sequence typing and single nucleotide polymorphism analysis of the core genome confirmed the diverse phylogeny of the strains. The analysis also revealed a close phylogenetic relationship between the EAEC O104:H4 strains in this outbreak and the strain of *E. coli* O104:H4 associated with a large outbreak of haemolytic ureamic syndrome in Germany in 2011. Further analysis of the EAEC plasmids, encoding the key enteroaggregative virulence genes, showed diversity with respect to FIB/FII type, gene content and genomic architecture. Known EAEC virulence genes, such as *aggR, aat* and *aap*, were present in all but one of the strains. A variety of fimbrial genes were observed, including genes encoding all five known fimbrial types, AAF/1 to AAF/V. The AAI operon was present in its entirety in 15 of the EAEC strains, absent in three and present, but incomplete, in two isolates. EAEC is known to be a diverse pathotype and this study demonstrates that a high level of diversity in strains recovered from cases associated with a single outbreak. Although the EAEC in this study did not carry the *stx* genes, this outbreak provides further evidence of the pathogenic potential of the EAEC O104:H4 serotype.

## Introduction

The Enteroaggregative *Escherichia coli* (EAEC) group is a large, diverse group of diarrhoeagenic *E. coli* originally defined by their adherence to HEp-2 cells in a stacked brick formation [1]. Generally, EAEC are detected and identified using PCR targeting EAEC associated virulence genes that are predominately plasmid encoded, including a regulator of multiple plasmid virulence factors (*aggR*), the anti-aggregation transporter gene (*aat*) and the gene encoding dispersin (*aap*) [2]–[4]. AggR also activates the expression of the chromosomal *aai* genes encoding a Type VI Secretion System (T6SS) [5].

Virulence gene content associated with EAEC is highly variable between different strains, as illustrated in studies aimed at genotyping EAEC from a variety of clinical sources, healthy control groups and outbreaks [6]–[9]. In these studies, strains show inconsistent presence and concordance of EAEC virulence genes by PCR in specimens from symptomatic and asymptomatic cases. These data suggest that the full genetic component of this phenotype is not yet fully understood and, although most of these genes are found on the aggregative virulence plasmid, their inheritability is complex.

Early research on EAEC linked these strains to persistent diarrhoea in children in developing countries but EAEC have since been shown to be a significant cause of acute diarrhoea and important in the aetiology of intestinal infections in industrialized countries [10]. Two independent, large, prospective studies of diarrhoea aetiology conducted in the UK (1993–1996) and USA (2002–2004) reported a similar EAEC prevalence in patients with diarrhoea: 4.6% (160/3506) and 4.5% (37/823) in the UK and US studies, respectively, and 1.7% in control subjects from both studies [11], [12]. Clinical symptoms include watery diarrhoea, often with mucus, low grade fever, abdominal pain, nausea and vomiting [10].

Several EAEC foodborne outbreaks of gastroenteritis have been documented, notably in Japan, the UK and Italy [13]–[15]. Recently, a strain of enteroaggregative Shiga toxin-producing *E. coli* O104:H4 was identified as the cause of a foodborne outbreak of bloody diarrhoea and haemolytic ureamic syndrome (HUS) in Germany and France [16]–[20]. Case-control, cohort and trace back studies implicated fenugreek sprouts from Egypt as the source of the infection [21]. Detailed and timely microbiological outbreak investigations were followed by whole genome sequencing of strains of *E. coli* O104:H4 by various international groups [17], [19], [22]–[23].

In March 2013, a large outbreak of GI disease occurred in the North East of England and cases were linked to a food festival. Four hundred and thirteen cases reported illness including symptoms of persistent diarrhoea and abdominal pain immediately following the event, and a total of 592 cases were identified following an on-line questionnaire. One hundred and ten specimens were submitted to the regional Public Health England and local hospital laboratories. Using traditional culture methods, *Salmonella enterica* serotype Agona was isolated from 25 cases and 4 further cases had other *Salmonella* species. Cohort and trace back studies implicated a contaminated, fresh curry leaves from Pakistan as the source of the infection.

The low number of cases testing positive for *Salmonella* species raised the suspicion that this was a multi-pathogen outbreak and further testing using a pan pathogen PCR was requested by the Outbreak Control Team. Subsequently, strains of EAEC harbouring *aggR* were isolated from PCR positive faecal specimens. The aim of this study was to use whole genome sequencing to explore the genomic diversity of the 20 strains of EAEC harbouring *aggR* by determining their phylogenetic relationship, plasmid type and virulence gene content and to assess the likely contribution of each strain type to the reported symptoms of GI disease.

## Material and Methods

### Microbiology

Retrospectively, 88 faecal specimens from cases associated with the outbreak were tested for the presence of other bacterial GI pathogens using a multiplex GI pathogens PCR [24]. Although the faecal specimens had been stored for over 10 weeks at 4°C, an attempt was made to isolate the pathogens detected by the multiplex PCR by testing individual colonies for the *stx*, *ipaH* and *aggR* target genes, associated with Shiga toxin-producing *Escherichia coli* (STEC), *Shigella* species and EAEC respectively. For faecal specimens positive for *aggR*, 20 colonies were picked from bacterial growth on MacConkey or Sorbitol MacConkey agar plates and retested using the same PCR. Those colonies harbouring the *aggR* genes were identified biochemically as *E. coli* and serotyped using antisera raised in rabbits to the *E. coli* somatic O antigens.

### Library preparation and whole genome sequencing

DNA was extracted for sequencing using the Wizard kit (Promega UK). Paired-end libraries were generated using the Illumina Nextera XT sample preparation kit. Automated platforms were used for sample preparation, library generation and quality checks. Assessment of fragment sizes was performed on the Perkin Elmer Labchip GX after fragmentation and clean-up. After normalisation, samples were pooled by hand and library quantification was performed using the KAPA library quantification kit for Illumina sequencing, on an ABI Viia7. Libraries were diluted to 15 pM and denatured at 96°C on a heat block for 2 minutes before being placed on ice for 5 minutes. Denatured libraries were spiked with 5% phiX and loaded on a Rapid flowcell by the Illumina cBot instrument. Paired-end sequencing was performed on the Illumina HiSeq 2500 instrument running HCS 2.0.10.0 using the TruSeq Rapid SBS kit (200 cycle) and TruSeq Paired-end rapid cluster kit. The following cycle parameters were used for sequencing: Read 1: 101, Index read 1: 8, Index read 2: 8 and Read 2: 101. RTA version 1.17.21.3 was used for generation of base call files.

Spades version 2.5.1 [25] was used to produce *de novo* assemblies of the sequenced paired-end fastq file. The number of contigs produced ranged from 221 to 552 per sample with N50s from 48338 to 192731 nucleotides.

### Phylogenetic analysis

Illumina reads were mapped to the reference EAEC strain 55989 using BWA-SW [26]. The Sequence Alignment Map output from BWA was sorted and indexed to produce a Binary Alignment Map (BAM) using Samtools [27]. GATK2 [28] was used to create a Variant Call Format (VCF) file from each of the BAMs, which were further parsed to extract only single nucleotide polymorphism (SNP) positions which were of high quality in all genomes (MQ>30, DP>10, 128 GQ>30, Variant Ratio >0.9). Pseudosequences of polymorphic positions were used to create approximate maximum likelihood trees using FastTree [29] under the General time reversible (GTR) model of nucleotide evolution.

### Multilocus sequence typing (MLST)

MLST types were identified by mapping the reads against all *E. coli* allele variants held in the Achtman MLST database (www.mlst.ucc.ie/mlst/dbs/Ecoli) using a modification of the SRST software [30].

### Plasmid FIB/FII typing

Plasmid incompatibility groups were determined using the specific sequences for plasmid replicon types defined by Carattoli *et al*. 2005 [31]. These sequences were searched for using blastn against the assembled genomes. Retrieved IncF and IncI replicon sequences were extracted *in silico* and further characterised to sequence type level according to the new scheme described in the plasmid MLST database (pMLST: www.pubmlst.or/plasmid/)

### BRIG analysis

Assembled genomes were loaded into BRIG as concentric rings [32] and compared against the pAA reference genome using blastn. pAA annotations from genbank file were added in the final ring.

### Mapping of known EAEC virulence genes

Illumina reads were mapped to a panel of putative EAEC virulence factors (*aggR, aatA, aatB, aatC, aatP, aap, SepA, IDI* and *aaiC*) using BWA-SW [26]. The number of reads that mapped to each position was calculated using Samtools mpileup [27]. The aggregative fimbrial adhesion type was determined based on mapping to each of the five variable fimbrial subunits AAF/I to AAFV (*aggA, aafA, agg3a, hdaA, aaf5a*) [33]–[37].

### Determination of the presence or absence of AAI operon T6SS components using BLAST

AAI operon T6SS coding genes were extracted from the reference strain 55989 genbank file (http://www.ncbi.nlm.nih.gov/nuccore/NC_011748.1) and made into a BLAST database. Each of the assembled genomes was queried against the database using blastn to recover whether it had significant hits for each component of the AAI.

### Data Submission

The short read sequence data has been deposited in the NCBI Short Read Archive under the BioProject PRJNA245029

## Results

### Detection of multiple GI pathogens by PCR

Retrospectively, 88 specimens from cases associated with the outbreak were tested for the presence of other bacterial GI pathogens using a multiplex GI pathogens PCR [24]. A variety of bacterial GI pathogens were detected by PCR from 88 of the stored faecal specimens from cases associated with the outbreak including *Salmonella* (3 cases), STEC (5 cases) and *Shigella* (29 cases). The *aggR* gene was identified in 65 (75%) specimens. Twenty strains of EAEC harbouring *aggR* were isolated from the 65 PCR positive faecal specimens. No STEC or *Shigella* species were isolated.

### Phylogeny of EAEC isolated from the cases associated with the outbreak

Ten different serotypes and nine MLSTs were identified among the EAEC isolated from the outbreak cases (Table 1). The most commonly observed serotypes were O131:H27 (6), O104:H4 (5) and O20:H19 (2), and the most frequently identified STs, corresponding with these serotypes, were ST10, ST678 and ST278 respectively.

**Table 1:** Phenotypic and genotypic characteristic of the 20 strains of EAEC isolated from faecal specimens linked to the outbreak.

Isolate Number	Isolate ID	Serotype	MLST	Plasmid type	Fimbrial type	Antibiotic resistance profile(ESBL *)
1	216/13	O104:H4	678	FIB_25 FII_48	I	AMP/SUL/STR/TET/TMP/NAL
2	218/13	O104:H4	678	FIB_25 FII_48	I	AMP/SUL/STR/TET/TMP/NAL
3	1062/13	O104:H4	678	FIB_25 FII_48	I	AMP/SUL/STR/TET/TMP/NAL
4	1063/13	O104:H4	678	FIB_25 FII_48	I	AMP/SUL/STR/TET/TMP/NAL
5	1070/13	O104:H4	678	FIB_25 FII_48	I	AMP/SUL/STR/TET/TMP/NAL
6	0219/13	O131:H27	10	FIB_5 FII_17	I	AMP/SUL/STR/TET/TMP/NAL
7	0220/13	O131:H27	10	FIB_5 FII_17	I	AMP/SUL/STR/TET/TMP/NAL
8	1071/13	O131:H27	10	FIB_5 FII_17	I	AMP/SUL/STR/TET/TMP/NAL
9	1072/13	O131:H27	10	FIB_5 FII_17	I	AMP/SUL/STR/TET/TMP/NAL
10	1073/13	O131:H27	10	FIB_5 FII_17	I	AMP/SUL/STR/TET/TMP/NAL
11	1074/13	O131:H27	10	FIB_5 FII_17	I	AMP/SUL/STR/TET/TMP/NAL
12	0215/13	O20:H19	278	FIB_5 FII_17	IV	AMP/SUL/TMP/CAZ/CTX/CPR/CEF *
13	0221/13	O20:H19	278	FIB_5 FII_17	IV	AMP/SUL/TMP/CAZ/CTX/CPR/CEF *
14	0222/13	O19a:H30	38	FIB_33 FII_1	III	AMP/SUL/STR/TMP/NAL/CAZ/CTX/CEF
15	1061/13	O55:H19	10	FIB_33 FII_1	III	AMP/SUL/STR/TEM/NAL
16	1065/13	O63:H12	1664	FIB_33 FII_1	III	AMP/SUL/STR/TET/TMP/NAL/CIP/CAZ/CTX/CEF
17	0214/13	O?:H19	746	FII	III	NAL
18	0217/13	O33:H16	295	FII_9	II	AMP/CHL/STR/TET/TMP/NAL
19	1064/13	O?:H21	227	FII_9	II	AMP/SUL/STR/TET/TMP/NAL/CAZ/CTX/CPR/CEF *
20	1060/13	O111:H4	226	repB	-	AMP/CHL/COL/TET/NAL

Key: AMP Ampicillin; CAZ ceftazidime; CTX Cefataxime; CEF Ceftiofur; CPR cefpirome; NAL nalidixic acid; STR Streptomycin; SUL Sulphonamide; TET tetracycline; TMP Trimethoprim.

**Figure 1:**
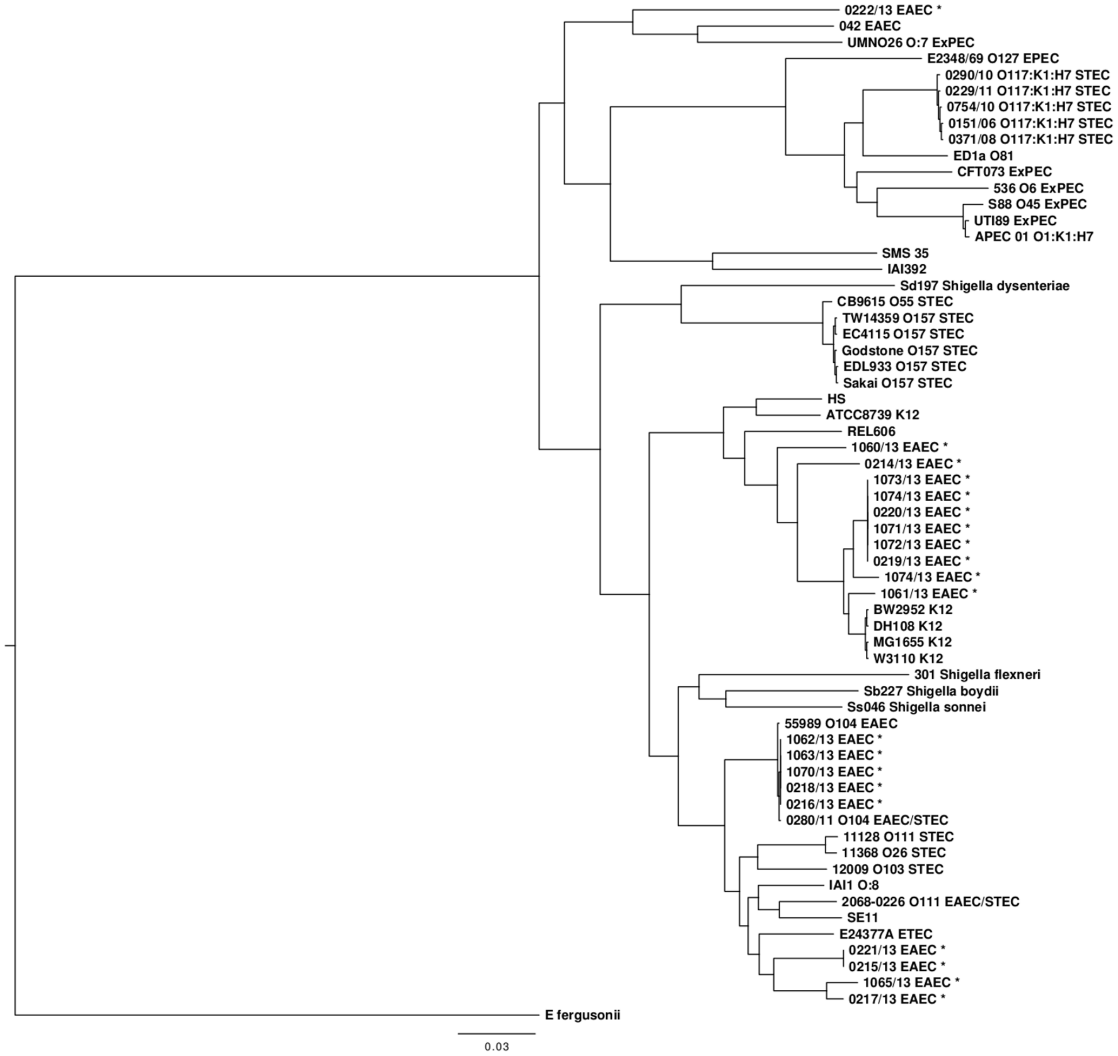
Whole genome chromosomal phylogeny of strains of *E. coli* and *Shigella* spp using previously published sequences and showing the 20 strains of EAEC isolates during this outbreak (highlighted in red). EAEC Enteroaggregative *E. coli*; EHEC Enterohaemorrhagic *E. coli*; ExPEC Extraintestinal Pathogenic *E. Coli*; EPEC Enteropathogenic *E. coli*; ETEC Enterotoxigenic *E. coli*.

**Figure 2:**
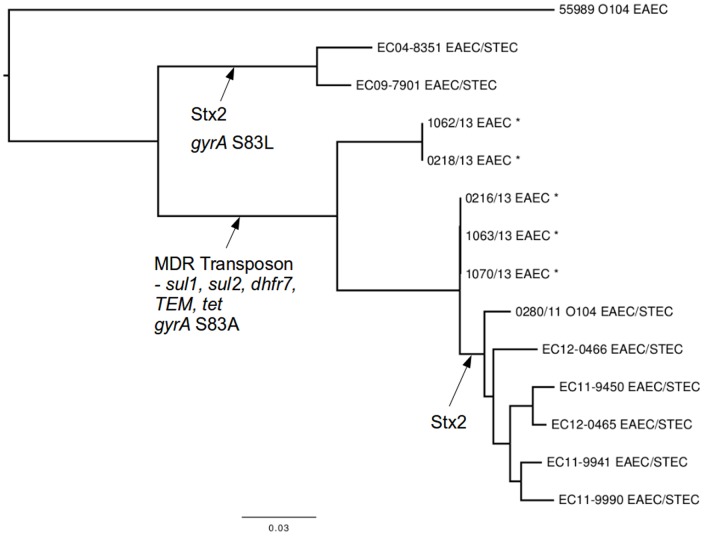
Chromosomal phylogeny of sequenced EAEC ST687 (strains including five strains isolated during this study marked *) represented as a maximum-likelihood tree. Previously published genome sequences included 280/11 isolated from a case linked to the outbreak in Germany in 2011 and 55989 isolated in the late 1990s in the Central African Republic. Other strains were previously described in Grad *at al*. 2013.

SNP analysis confirmed that the strains were phylogenetically diverse between serotypes (Figure 1). Strains belonging to the same serotype clustered on the same branch of the tree, however, even within the same serotype, isolates were phylogenetically distinct. Figure 2 shows a phylogeny based on 3115 core SNPs of 14 strains of *E. coli* O104:H4 and illustrates the relationship between the EAEC O104:H4 strains in this study with sporadic strains of *E. coli* O104:H4 and the strain associated with the *E. coli* O104:H4 outbreak in Germany in 2011 [22], [23]. Although none of the EAEC O104:H4 isolates in this study had the *stx* gene they share a common ancestor with the German outbreak strain 280/11 and the sporadic *stx* harboring enteroaggregative strains characterised by Grad *et al* (23). All five strains of *E. coli* O104:H4 isolated during this study share the MDR genomic island conferring resistance to ampicillin, the sulphonamides, streptomycin and tetracycline, and the S83A gyrA mutation in common with German outbreak strain and the closely related EAEC/STEC sporadic isolates from France. The EAEC O104:H4 strains isolated in this study are phylogenetically integrated with strains of EAEC/STEC suggesting either multiple gain or gain then loss of the *stx* phage within the O104:H4 serotype.

None of the EAEC O104:H4 isolates in this study had the *stx* gene or carried the extended spectrum beta lactamase (ESBL) plasmid characteristic of the 280/11 strain, although three other strains isolated during this study were identified phenotypically and genotypically as being ESBL-producers (Table 1).

### Replicon types of the EAEC plasmids encoding the key enteroaggregative virulence genes (pEAEC)

Multiple replicon types were observed with multiple combinations of FII and FIB proteins, with all but three plasmids having both the FIB and FII replicon types (Table1). Plasmids of type FIB5_FII17 were carried by strains belonging to two serotypes, O131:H27 and O20:H19. The plasmid type FIB25_FII48 harboured by the strains of EAEC O104:H4 was the same FIB/FII type described in the strains of *E. coli* O104:H4 linked to the 2011 German outbreak (Table 1).

### pEAEC encoded virulence genes and genomic architecture

**Figure 3:**
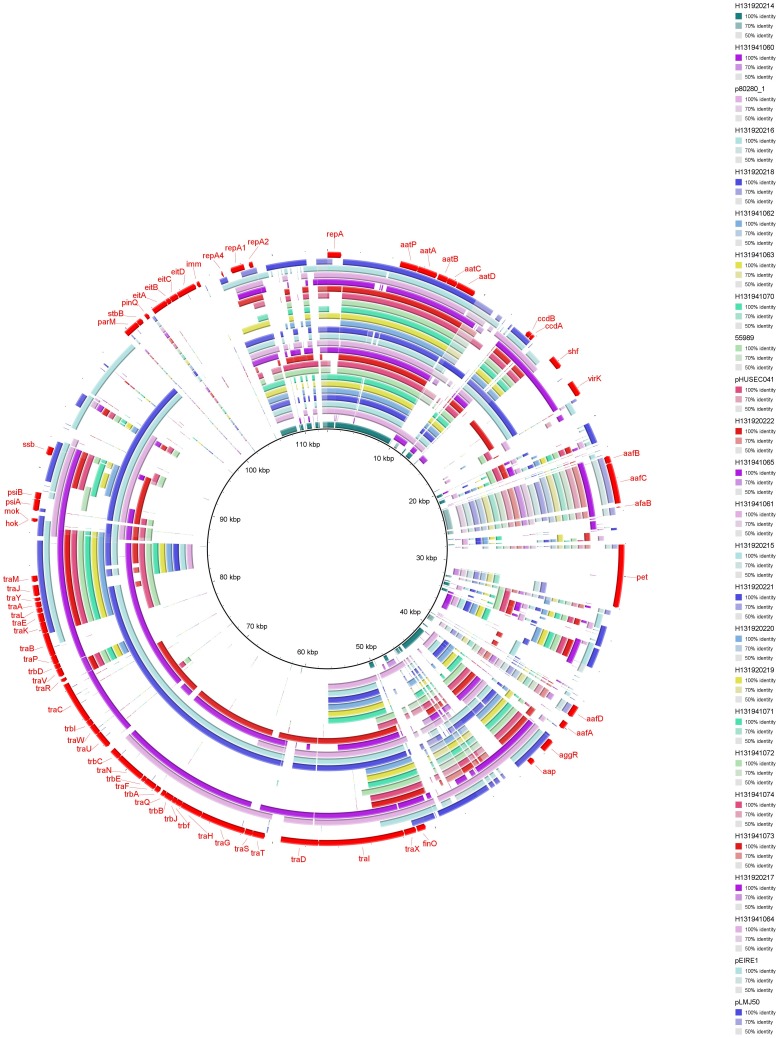
Assembled genomes displayed as concentric rings using BRIG and BLASTed against pAA genbank file as a reference. Coloured bars represent regions of homology. The darker shades represent a high percentage similarity, lighter shades represent lower levels of similarity and the absence of colour signifies absence of the gene.

Several plasmid encoded genes associated with EAEC have been described in previous studies. These include the transcriptional activator *aggR*, the anti-aggregation transporter locus *aat*, the anti-aggregative dispersin protein *aap* (2–4), the aggregative adherence fimbriae (AAF) (30–34), the serine protease autotransporter toxin *SepA* [38] and the recently described putative isopentenyl isomerase (IDI) enzymes [39]. Table 2 shows the number of reads that mapped to these targets in each outbreak isolate. All of the strains, apart from *E. coli* O111:H4 designated 1060/13, had sequence reads that mapped to *aggR, aat, aap* and the putative IDI enzymes. This isolate originally tested positive with the *aggR* PCR subsequently tested negative following storage on Dorset Egg medium at room temperature. It is likely that this isolate lost the EAEC plasmid during storage. The serine protease *sepA* was present in 16 of the 20 EAEC strains isolated from the cases associated with the outbreak. Whilst the pEAEC virulence gene complement was conserved, the genomic context in terms of flanking IS elements was highly variable across the different plasmids (Figure 3).

**Table 2:** Number of mapped reads to the EAEC virulence genes in each outbreak isolate.

Isolate Number	Isolate ID	Serotype	AggR	aatA	aap	sepA	IDI	aaiC
1	216/13	O104:H4	1001	759	752	387	631	1780
2	218/13	O104:H4	1752	1783	874	443	1278	2213
3	1062/13	O104:H4	1932	1593	1525	805	1360	2195
4	1063/13	O104:H4	1483	1145	1133	733	1056	1165
5	1070/13	O104:H4	1554	1345	866	434	897	3219
6	0219/13	O131:H27	1799	799	540	128	591	2335
7	0220/13	O131:H27	1792	739	563	172	584	2683
8	1071/13	O131:H27	3501	1705	1054	347	1123	2228
9	1072/13	O131:H27	5185	2300	1607	366	1472	5146
10	1073/13	O131:H27	3337	1599	973	273	999	1879
11	1074/13	O131:H27	5160	2455	1357	378	1566	1943
12	0215/13	O20:H19	1826	1545	831	271	781	1841
13	0221/13	O20:H19	757	615	366	136	264	1390
14	0222/13	O19a:H30	685	568	493	118	393	**0**
15	1061/13	O55:H19	1813	1859	1206	355	1127	1598
16	1065/13	O63:H12	2339	2313	1448	383	1558	**2**
17	0214/13	O?:H19	2380	1963	1901	**32**	1533	**270**
18	0217/13	O33:H16	794	620	716	**10**	827	2842
19	1064/13	O?:H21	3008	3045	2159	**1**	2291	**0**
20	1060/13	O111:H4	**0**	**0**	**0**	**0**	**0**	**193**

Where the average coverage is less than 10% across the length of the gene the cell in the table is highlighted in bold.

Five types of pEAEC associated AAF have been described [33]–[37] and all five fimbriae types were identified in the strains analysed during this study. Strains of EAEC belonging to serotypes O104:H4 and O131:H27 had AAF/I fimbriae, as seen in the aggregative plasmid of *E. coli* O104:H4 linked to the 2011 German outbreak. Those strains belonging to serotype O20:H19 had the Type IV fimbriae (HdaA) [36]. AAF/II, AAF/III and AAF/V fimbriae were detected in five strains belonging to five different serotypes but three harbouring the same plasmid type, FIB33_FII1 (Table 2).

### AAI operon encoding the putative T6SS

A 117 kb pathogenicity island, first described in the chromosome of EAEC 042, has been implicated as an EAEC pathogenicity factor. Twenty-five contiguous genes (*aaiA–Y*) in this island were previously shown to be transcriptionally activated by the plasmid encoded AggR protein and encoded for a T6SS [5]. In the EAEC strains isolated from the outbreak cases described in this study, the AAI operon was present in its entirety in the strains belonging to serotypes O104:H4, O131:H27, O20:H19 and O55:H19, whilst the island was absent in the strains belonging to the serotypes O19a:H30, O?:H21 and O63:H12 (Figure 3). Table 3 shows the distribution of the putative T6SS genes, *aaiA* to *aaiN*, in the outbreak strains. In the EAEC strains designated 1060/13 and 0214/13 (serotypes O111:H4 and O?:H19 respectively), a contig with 84% identity to the AAI operon and no homology to the NCBI non-redundant database was identified. In the EAEC O?:H19 isolate this homologue to *aai* was co-located on a contig with a plasmid addiction system suggestive of a non-chromosomal location in these strains.

**Table 3:** The distribution of the putative T6SS genes *aaiA* to *aaiN* in the outbreak strains.

Serotypes									
Gene	O104:H4	O33:H27	O20:H19	O55:H19	O111:H4	O?:H19	O19a:H30	O63:H12	O?:H21
aaiA	X	X	X	X	X	X			
aaiB	X	X	X	X	X	X			
aaiC	X	X	X	X					
aaiD	X	X	X	X	X	X			
aaiE	X	X	X	X	X	X			
aaiF	X	X	X	X	X	X			
aaiG	X	X	X	X	X	X			
aaiH	X	X	X	X					
aaiI	X	X	X	X					
aaiJ	X	X	X	X	X	X			
aaiK	X	X	X	X	X	X			
aaiL	X	X	X	X	X	X			
aaiM	X	X	X	X	X	X			
aaiN	X	X	X	X	X	X			

X indicates the genes was present on the genome.

## Discussion

Historically, outbreaks have been associated with strains of a single pathogen exhibiting similar, if not identical, phenotypic and genotypic characteristics. However, the multiplex PCR approach to detection of GI pathogens directly from faecal specimens has provided good evidence that many individual cases of diarrhoea and outbreaks of GI disease are associated with multiple pathogens [40]–[41]. Although there was clear microbiology evidence that established GI pathogens, such as *Salmonella* and *Shigella* species, played a significant part, the symptoms described by the cases and the presence of *aggR* in 75% of the specimens retrospectively tested by PCR, suggested that certain serotypes of the EAEC isolated, contributed to the GI disease associated with this outbreak. However, the variety of EAEC serotypes identified in the 20 strains isolated presented a complex picture.

Initially, it was suggested that the variation in serotype in the outbreak strains was masking a closer phylogenetic relationship. However, the phylogenetic tree created by comparing SNPs in the core genome showed that, although strains of the same serotype were relatively closely related, those of different serotypes were diverse. EAEC belong to several lineages with different evolutionary histories demonstrating independent acquisition of the plasmids encoding EAEC virulence genes [42]. Conversely, strains with a recent common ancestor, e.g. those that share an MLST sequence type, may have different pathotypes [43]. For example, *E. coli* O104:H4 ST678 has been shown to be STEC and EAEC [19]. The pathotype distribution is explained with multiple loss/gain events of pathogenicity elements.

Although strains of EAEC have been shown to harbour a wide diversity of plasmids that encode the enteroaggregative phenotype even in conserved chromosomal backgrounds [19], it was considered possible that similar plasmids would be found in the different strains of EAEC linked to this outbreak, given their spatial and temporal association. However, analysis of the plasmid genomes showed that they demonstrated a high level of variation in replicon type, gene content and genomic architecture. Some plasmid similarity was seen within strains of the same MLST and serotype but wide diversity was observed between different MLST and serotypes. The interspersing of different plasmids in the phylogeny suggests that the aggregative phenotype (specifically the presence of *aggR, aat* and *aap*) has been acquired by several different replicons of F-plasmids on multiple occasions. This level of strain and plasmid diversity has not previously been identified in isolates of EAEC from the same outbreak, although EAEC outbreaks involving more than one serotype and variation in pEAEC have been described previously [14], [44]

Generally, *aggR, aat* and *aap* were conserved between strains of EAEC linked to this outbreak but a variety of fimbrial genes were identified. The presence of AAF is required for mediating the aggregative adherence seen in EAEC. To date five non-homologous AAF fimbiral structural proteins have been described and a representative of each was identified in strains belonging to this outbreak.

*aaiA-P* comprise a T6SS apparatus for *aaiC* and was the first example of a conserved chromosomal aggregative genotype whose expression is under the control of a conserved plasmid encoded pathogenicity factor *AggR* [5]. In this study, five isolates all harbouring *aggR* and *aat*, had a missing or an incomplete AAI operon. This raises a question regarding the pathogenic potential of the *aggR*-positive but AAI operon deficient strains, in relation to those *aggR*-positive strains with complete AAI cassettes. Previous prevalence studies detecting the *aaiA* show its presence in between 26 and 44% of phenotypic aggregative *E. coli* [45], [46]. Animal models for investigating EAEC virulence have been described previously [47] and further virulence studies are required. Interestingly, two strains (EAEC O111:H4 and O?:H19) had incomplete AAI operons encoding a T6SS. These regions were homologous to those found in serotypes O104:H4, O131:H27, O20:H19 and O55:H19 but with a different *aaiC* component. In addition, there was some evidence that it may be plasmid-encoded.

It was suggested that certain strains of EAEC may have been carried asymptomatically by the cases before the outbreak occurred. Hwoever, it was not possible to compare the serotypes isolates following the outbreak with the serotypes of strains of EAEC currently circulating in England as there are very little data on domestically acquired strains of EAEC. Surveillance data indicates that the majority of strains of EAEC isolate in England are from cases of travellers' diarrhoea [12], [50].

One hypothesis is that not all the strains of EAEC associated with this outbreak had the same level of pathogenicity and that only certain EAEC serotypes isolated contributed to the symptoms described. For example, a complete AAI operon may increase the pathogenic potential of strains of *E. coli* harbouring the pEAEC. Other studies have suggested that strains harbouring different fimbrial types maybe more pathogenic that others. Nüesch-Inderbinen *et al*. (2013) [48] showed a statistically significant association of the agg3C gene with the asymptomatic state. The presence of AAF/I and AAF/II have been associated with symptomatic cases [6], [49].

Importantly, although colonies of EAEC O104:H4 were isolated from only five outbreak cases, all faecal specimens were retrospectively tested by PCR for the presence of the O104 O-antigen gene (*wzx*O104) [51]. The PCR detected *wzx*O104 in faecal specimens from 36 cases. The EAEC serotype O104:H4, with and without *stx2*, has been previously identified as a cause of GI disease. Furthermore, although the EAEC O104:H4 in this study did not carry the *stx* genes, this outbreak provides further evidence of the pathogenic potential of this EAEC serotype. EAEC is known to be a diverse pathotype and this study demonstrates this diversity can be seen within a single outbreak.

## References

[1] Nataro JP, Kaper JB, Robins-Browne R, Prado V, Vial P, et al (1987) Patterns of adherence of diarrheagenic *Escherichia coli* to HEp-2 cells. Pediatr Infect Dis J 6: 829–31. 3313248 10.1097/00006454-198709000-00008

[2] Nataro JP, Yikang D, Yingkang D, Walker K (1994) AggR, a transcriptional activator of aggregative adherence fimbria I expression in enteroaggregative *Escherichia coli* . J Bacteriol 176: 4691–9. 7913930 10.1128/jb.176.15.4691-4699.1994 PMC196291

[3] Sheikh J, Czeczulin JR, Harrington S, Hicks S, Henderson IR, et al (2002) A novel dispersin protein in enteroaggregative *Escherichia coli* . J Clin Invest 110: 1329–37. 12417572 10.1172/JCI16172 PMC151617

[4] Nishi J, Sheikh J, Mizuguchi K, Luisi B, Burland V, et al (2003) The export of coat protein from enteroaggregative *Escherichia coli* by a specific ATP-binding cassette transporter system. J Biol Chem 278: 45680–9. 12933818 10.1074/jbc.M306413200

[5] Dudley EG, Thomson NR, Parkhill J, Morin NP, Nataro JP (2006) Proteomic and microarray characterization of the AggR regulon identifies a pheU pathogenicity island in enteroaggregative *Escherichia coli* . Mol Microbiol 61: 1267–82. 16925558 10.1111/j.1365-2958.2006.05281.x

[6] Jenkins C, Chart H, Willshaw GA, Cheasty T, Tompkins DS (2007) Association of putative pathogenicity genes with adherence characteristics and fimbrial genotypes in typical enteroaggregative Escherichia coli from patients with and without diarrhoea in the United Kingdom. Eur J Clin Microbiol Infect Dis 26: 901–6. 17899229 10.1007/s10096-007-0388-z

[7] Jenkins C, Tembo M, Chart H, Cheasty T, Willshaw GA, et al (2006a) Detection of enteroaggregative *Escherichia coli* in faecal samples from patients in the community with diarrhoea. J Med Microbiol 55: 1493–7. 17030907 10.1099/jmm.0.46683-0

[8] Boisen N, Scheutz F, Rasko DA, Redman JC, Persson S, et al (2012) Genomic characterization of enteroaggregative *Escherichia coli* from children in Mali. J Infect Dis 205: 431–44. 22184729 10.1093/infdis/jir757 PMC3256949

[9] Okeke IN, Wallace-Gadsden F, Simons HR, Matthews N, Labar AS, et al (2010) Multi-locus sequence typing of enteroaggregative *Escherichia coli* isolates from Nigerian children uncovers multiple lineages. PLoS One 5: e14093. 21124856 10.1371/journal.pone.0014093 PMC2990770

[10] Okeke IN, Nataro JP (2001) Enteroaggregative *Escherichia coli* Lancet Infect Dis 1(5): :304–13. Review 10.1016/S1473-3099(01)00144-X 11871803

[11] Nataro JP, Mai V, Johnson J, Blackwelder WC, Heimer R, et al (2006) Diarrheagenic *Escherichia coli* infection in Baltimore, Maryland, and New Haven, Connecticut. Clin Infect Dis 43: 402–7. 16838226 10.1086/505867

[12] Wilson A, Evans J, Chart H, Cheasty T, Wheeler JG, et al (2001) Characterisation of strains of enteroaggregative *Escherichia coli* isolated during the infectious intestinal disease study in England. Eur J Epidemiol 17: 1125–30. 12530772 10.1023/a:1021224915322

[13] Itoh Y, Nagano I, Kunishima M, Ezaki T (1997) Laboratory investigation of enteroaggregative *Escherichia coli* O untypeable:H10 associated with a massive outbreak of gastrointestinal illness. J Clin Microbiol 35: 2546–50. 9316905 10.1128/jcm.35.10.2546-2550.1997 PMC230008

[14] Smith HR, Cheasty T, Rowe B (1997) Enteroaggregative *Escherichia coli* and outbreaks of gastroenteritis in UK. Lancet 350: 814–5. 10.1016/s0140-6736(05)62611-6 9298029

[15] Scavia G, Staffolani M, Fisichella S, Striano G, Colletta S, et al (2008) Enteroaggregative *Escherichia coli* associated with a foodborne outbreak of gastroenteritis. J Med Microbiol 7: 1141–6. 10.1099/jmm.0.2008/001362-0 18719185

[16] Frank C, Werber D, Cramer JP, Askar M, Faber M, et al (2011) Epidemic profile of Shiga-toxin-producing *Escherichia coli* O104:H4 outbreak in Germany. N Engl J Med 365: 1771–80. 21696328 10.1056/NEJMoa1106483

[17] Mellmann A, Harmsen D, Cummings CA, Zentz EB, Leopold SR, et al (2011) Prospective genomic characterization of the German enterohemorrhagic *Escherichia coli* O104:H4 outbreak by rapid next generation sequencing technology. PLoS One 6: e22751. 21799941 10.1371/journal.pone.0022751 PMC3140518

[18] Bielaszewska M, Mellmann A, Zhang W, Köck R, Fruth A, et al (2011) Characterisation of the *Escherichia coli* strain associated with an outbreak of haemolytic uraemic syndrome in Germany, 2011: a microbiological study. Lancet Infect Di 11: 671–6. 10.1016/S1473-3099(11)70165-7 21703928

[19] Rasko DA, Webster DR, Sahl JW, Bashir A, Boisen N, et al (2011) Origins of the *E. coli* strain causing an outbreak of hemolytic-uremic syndrome in Germany. N Engl J Med 365: 709–17. 21793740 10.1056/NEJMoa1106920 PMC3168948

[20] King LA, Nogareda F, Weill FX, Mariani-Kurkdjian P, Loukiadis E, et al (2012) Outbreak of Shiga toxin-producing Escherichia coli O104:H4 associated with organic fenugreek sprouts, France, June 2011. Clin Infect Dis 54: 1588–94. 22460976 10.1093/cid/cis255

[21] Buchholz U, Bernard H, Werber D, Böhmer MM, Remschmidt C, et al (2011) German outbreak of *Escherichia coli* O104:H4 associated with sprouts. N Engl J Med 365: 1763–70. 22029753 10.1056/NEJMoa1106482

[22] Grad YH, Lipsitch M, Feldgarden M, Arachchi HM, Cerqueira GC, et al (2012) Genomic epidemiology of the *Escherichia coli* O104:H4 outbreaks in Europe, 2011. Proc Natl Acad Sci U S A 109: 3065–70. 22315421 10.1073/pnas.1121491109 PMC3286951

[23] Grad YH, Godfrey P, Cerquiera GC, Mariani-Kurkdjian P, Gouali M, et al (2013) Comparative genomics of recent Shiga toxin-producing Escherichia coli O104:H4: short-term evolution of an emerging pathogen. MBio 4: e00452–12. 23341549 10.1128/mBio.00452-12 PMC3551546

[24] Moran-Gilad J, Chand M, Brown C, Shetty N, Morris G, et al (2012) Microbiological aspects of public health planning and preparedness for the 2012 Olympic Games. Epidemiol Infect 140: 2142–51. 22892344 10.1017/S0950268812001835 PMC9152329

[25] Bankevich A, Nurk S, Antipov D, Gurevich AA, Dvorkin M, et al (2012) SPAdes: a new genome assembly algorithm and its applications to single-cell sequencing. J Comput Biol 19: 455–77. 22506599 10.1089/cmb.2012.0021 PMC3342519

[26] Li H, Durbin R (2010) Fast and accurate long-read alignment with Burrows-Wheeler transform. Bioinformatics 26: 589–95. 20080505 10.1093/bioinformatics/btp698 PMC2828108

[27] Li H, Handsaker B, Wysoker A, Fennell T, Ruan J, et al (2009) The Sequence Alignment/Map format and SAMtools. Bioinformatics 25: 2078–9. 19505943 10.1093/bioinformatics/btp352 PMC2723002

[28] McKenna A, Hanna M, Banks E, Sivachenko A, Cibulskis K, et al (2010) The Genome Analysis Toolkit: a MapReduce framework for analyzing next-generation DNA sequencing data. Genome Res 20: 1297–303. 20644199 10.1101/gr.107524.110 PMC2928508

[29] Price MN, Dehal PS, Arkin AP (2010) FastTree 2 - approximately maximum-likelihood trees for large alignments. PLoS One 5: e9490. 20224823 10.1371/journal.pone.0009490 PMC2835736

[30] Inouye M, Conwa TC, Zobel J, Holt KE (2012) Short read sequence typing (SRST): multi-locus sequence types from short reads. BMC Genomics 13: 338. 22827703 10.1186/1471-2164-13-338 PMC3460743

[31] Carattoli A, Bertini A, Villa L, Falbo V, Hopkins KL, et al (2005) Identification of plasmids by PCR-based replicon typing. J Microbiol Methods 63: 219–28. 15935499 10.1016/j.mimet.2005.03.018

[32] Alikhan NF, Petty NK, Ben Zakour NL, Beatson SA (2011). BLAST Ring Image Generator (BRIG): Simple prokaryote genome comparisons. *BMC genomics* 12: , 402. 10.1186/1471-2164-12-402 PMC3163573 21824423

[33] Nataro JP, Deng Y, Maneval DR, German AL, Martin WC, et al (1992) Infect Aggregative adherence fimbriae I of enteroaggregative *Escherichia coli* mediate adherence to HEp-2 cells and hemagglutination of human erythrocytes. Immun 60: 2297–304. 10.1128/iai.60.6.2297-2304.1992 PMC257157 1350273

[34] Czeczulin JR, Balepur S, Hicks S, Phillips A, Hall R, et al (1997) Aggregative adherence fimbria II, a second fimbrial antigen mediating aggregative adherence in enteroaggregative *Escherichia coli* . Infect Immun 65: 4135–45. 9317019 10.1128/iai.65.10.4135-4145.1997 PMC175595

[35] Bernier C, Gounon P, Le Bouguénec C (2002) Identification of an aggregative adhesion fimbria (AAF) type III-encoding operon in enteroaggregative *Escherichia coli* as a sensitive probe for detecting the AAF-encoding operon family. Infect Immun 70: 4302–11. 12117939 10.1128/IAI.70.8.4302-4311.2002 PMC128174

[36] Boisen N, Struve C, Scheutz F, Krogfelt KA, Nataro JP (2008) New adhesin of enteroaggregative *Escherichia coli* related to the Afa/Dr/AAF family. Infect Immun 76: 3281–92. 18443096 10.1128/IAI.01646-07 PMC2446688

[37] Dallman T, Smith GP, O'Brien B, Chattaway MA, Finlay D, et al (2012) Characterization of a verocytotoxin-producing enteroaggregative *Escherichia coli* serogroup O111:H21 strain associated with a household outbreak in Northern Ireland. J Clin Microbiol 50: 4116–9. 23035193 10.1128/JCM.02047-12 PMC3502983

[38] Boisen N, Ruiz-Perez F, Scheutz F, Krogfelt KA, Nataro JP (2009) Short report: high prevalence of serine protease autotransporter cytotoxins among strains of enteroaggregative *Escherichia coli* . Am J Trop Med Hyg 80: 294–301. 19190229 PMC2660206

[39] Morin N, Santiago AE, Ernst RK, Guillot SJ, Nataro JP (2013) Characterization of the AggR regulon in enteroaggregative *Escherichia coli* . Infect Immun 81: 122–32. 23090962 10.1128/IAI.00676-12 PMC3536136

[40] Tam CC, Rodrigues LC, Viviani L, Dodds JP, Evans MR, et al (2013) Burden and aetiology of diarrhoeal disease in infants and young children in developing countries (the Global Enteric Multicenter Study, GEMS): a prospective, case-control study. Gut 61: 69–77. 10.1016/S0140-6736(13)60844-2 23680352

[41] Kotloff KL, Nataro JP, Blackwelder WC, Nasrin D, Farag TH, et al (2013) Lancet. 382: 209–22. 10.1016/S0140-6736(13)60844-2 23680352

[42] Ogura Y, Ooka T, Iguchi A, Toh H, Asadulghani M, et al (2009) Comparative genomics reveal the mechanism of the parallel evolution of O157 and non-O157 enterohemorrhagic *Escherichia coli* . Proc Natl Acad Sci U S A 106: 17939–44. 19815525 10.1073/pnas.0903585106 PMC2764950

[43] Wirth T, Falush D, Lan R, Colles F, Mensa P, et al (2006) Sex and virulence in *Escherichia coli*: an evolutionary perspective. Mol Microbiol 60: 1136–51. 16689791 10.1111/j.1365-2958.2006.05172.x PMC1557465

[44] Spencer J, Smith HR, Chart H (1999) Characterization of enteroaggregative *Escherichia coli* isolated from outbreaks of diarrhoeal disease in England. Epidemiol Infect 123: 413–21. 10694151 10.1017/s0950268899002976 PMC2810774

[45] Jenkins C, Chart H, Willshaw GA, Cheasty T, Smith HR (2006b) Genotyping of enteroaggregative *Escherichia coli* and identification of target genes for the detection of both typical and atypical strains. Diagn Microbiol Infect Dis 55: 13–9. 16500068 10.1016/j.diagmicrobio.2005.10.019

[46] Jenkins C, van Ijperen C, Dudley EG, Chart H, Willshaw GA, et al (2005) Use of a microarray to assess the distribution of plasmid and chromosomal virulence genes in strains of enteroaggregative *Escherichia coli* . FEMS Microbiol Lett 253: 119–24. 16243450 10.1016/j.femsle.2005.09.040

[47] Hwang J, Mattei LM, VanArendonk LG, Meneely PM, Okeke IN (2010) A pathoadaptive deletion in an enteroaggregative *Escherichia coli* outbreak strain enhances virulence in a *Caenorhabditis elegans* model. Infect Immun 78: 4068–76. 20584976 10.1128/IAI.00014-10 PMC2937471

[48] Nüesch-Inderbinen MT, Hofer E, Hächler H, Beutin L, Stephan R (2013) Characteristics of enteroaggregative *Escherichia coli* isolated from healthy carriers and from patients with diarrhoea. Med Microbiol doi: 10.1099/jmm.0.065177-0. [Epub ahead of print] 10.1099/jmm.0.065177-0 24008499

[49] Okeke IN, Lamikanra A, Czeczulin J, Dubovsky F, Kaper JB (2000) Heterogeneous virulence of enteroaggregative *Escherichia coli* strains isolated from children in Southwest Nigeria. J Infect Dis 181: 252–60. 10608774 10.1086/315204

[50] Perry N, Jenkins C, Cheasty T, Wain J (2010) Diarrhoeagenic Escherichia coli from routine diagnostic faecal samples in England and Wales. J Med Microbiol 59: 870–2. 20378724 10.1099/jmm.0.018671-0

[51] Scheutz F, Nielsen EM, Frimodt-Møller J, Boisen N, Morabito S, et al. (2011) Characteristics of the enteroaggregative Shiga toxin/verotoxin-producing *Escherichia coli* O104:H4 strain causing the outbreak of haemolytic uraemic syndrome in Germany, May to June 2011. Euro Surveill 16(24).. 10.2807/ese.16.24.19889-en 21699770

